# Effect of a Polymercaptan Material on the Electro-Optical Properties of Polymer-Dispersed Liquid Crystal Films

**DOI:** 10.3390/molecules22010043

**Published:** 2016-12-30

**Authors:** Yujian Sun, Cuihong Zhang, Le Zhou, Hua Fang, Jianhua Huang, Haipeng Ma, Yi Zhang, Jie Yang, Lan-Ying Zhang, Ping Song, Yanzi Gao, Jiumei Xiao, Fasheng Li, Kexuan Li

**Affiliations:** 1 Department of Materials Physics and Chemistry, School of Materials Science and Engineering, University of Science and Technology Beijing, Beijing 100083, China; syj_anshan@sina.com (Y.S.); Fanghua0229@126.com (H.F.); jianhua_ustb@163.com (J.H.); 5896326123@163.com (Y.Z.); g20158323@xs.ustb.edu.cn (J.Y.); 2 Department of Materials Science and Engineering, College of Engineering, Peking University, Beijing 100871, China; luoqiudehudie@163.com (L.Z.); mahaipeng6666@163.com (H.M.); zhanglanying@pku.edu.cn (L.-Y.Z.); songping1985@163.com (P.S.); 3 Key Laboratory of Polymer Chemistry and Physics of Ministry of Education, Peking University, Beijing 100871, China; 4 Department of Applied Statistics and Science, Xijing University, Xi’an 710123, Shaanxi Province, China; 5 Department of Applied Mechanics, School of Mathematics and Physics, University of Science and Technology Beijing, Beijing 100083, China; 6 College of Medical Laboratory, Dalian Medical University, Dalian 116044, Liaoning Province, China

**Keywords:** polymer-dispersed liquid crystal, morphology, electro-optical properties, photopolymerization

## Abstract

Polymer-dispersed liquid crystal (PDLC) films were prepared by the ultraviolet-light-induced polymerization of photopolymerizable monomers in nematic liquid crystal/chiral dopant/thiol-acrylate reaction monomer composites. The effects of the chiral dopant and crosslinking agents on the electro-optical properties of the PDLC films were systematically investigate. While added the chiral dopant S811 into the PDLC films, the initial off-state transmittance of the films was decreased. It was found that the weight ratio among acrylate monomers, thiol monomer PETMP and the polymercaptan Capcure 3-800 showed great influence on the properties of the fabricated PDLC films because of the existence of competition between thiol-acrylate reaction and acrylate monomer polymerization reaction. While adding polymercaptans curing agent Capcure 3-800 with appropriate concentration into the PDLC system, lower driven voltage and higher contrast ratio were achieved. This made the polymer network and electro-optical properties of the PDLC films easily tunable by the introduction of the thiol monomers.

## 1. Introduction

Polymer-dispersed liquid crystal (PDLC) films that consist of micrometer-sized liquid crystal droplets dispersed in a polymer matrix are a kind of composite material [1,2,3]. PDLC films show transparent and light scattering states in the electric field-on and field-off states, respectively. In the electric field-off state, the refractive-index mismatch between the polymer matrix and the LC material gives rise to the light scattering of incident light. In the electric field-on state, the films become transparent because of the parallel alignment of the LC directors with the applied electric field if the refractive indices match between the LC droplets and polymer matrix [4]. The switching properties of the LC molecules within the domains depend on some variables, such as the size and shape of the domains, the molecular interactions between the LC material and the polymer matrix [5,6]. Unlike conventional liquid crystal (LC) displays, PDLC films are flexible, easy preparation, and there is no requirement for polarisers, alignment layers and precise control of the spacer between two substrates [7,8,9]. Recently, much attention has been devoted to PDLC systems due to their potential applications in electro-optical technology, such as switchable windows, flexible large-area displays, and other devices [10,11,12,13,14].

PDLCs can be fabricated by different methods, such as polymerization-induced phase separation (PIPS), thermally induced phase separation (TIPS), solvent-induced phase separation (SIPS) and microencapsulation process (MP) [15,16,17]. PIPS has been considered the most popular way, as it is quite convenient and allows for a high degree of control over the final properties of the PDLC films [18]. The matrixes in the PIPS can be cured from photo-initiated polymerization and thermally initiated polymerization monomers. The cure of the former can be finished in a few minutes at room temperature, whereas that of the latter is usually done for few hours at a higher temperature. In this situation, the PDLC films of which the matrixes made from photo-curable monomers have being attracted much attention.

To date, many researchers have focused on free radical systems based on (meth)acrylate monomers by the UV-initiated polymerization method [19], such as introducing fluorinated acrylates into PDLCs could reduce LC anchoring energy and subsequently lower switching voltage [20]. Until recently, Many research groups have studied allyl ether thiol-ene systems or vinyl ether thiol-ene systems for the polymer matrix to fabricate PDLC films [21,22], but there are no reports on acrylate monomer-thiol systems. Compared with the free radical systems based primarily on acrylates, the thiol-ene system has significant advantages: reduced oxygen polymerization, less light initiator dosage, excellent thermal insulation, high refractive index, inertness to oxidation and water resistance [23,24,25,26,27,28].

In this study, PDLC films were prepared by the ultraviolet-light-induced polymerization of photopolymerizable monomers in nematic liquid crystal/chiral dopant/thiol monomer composites. The effect of the chiral dopant and feed ratio of the thiol crosslinking agents on the electro-optical properties of the PDLC films was systematically investigate. It is important to mention that there were competition between acrylate monomer polymerization reaction and thiol-acrylate reaction due to the polymerization mechanism of thiol monomer. By optimizing the composition of samples, PDLC films with relative low driving voltage were achieved.

## 2. Results and Discussion

### 2.1. Morphology of the Polymer Network in the PDLC Films

**Figure 1:**
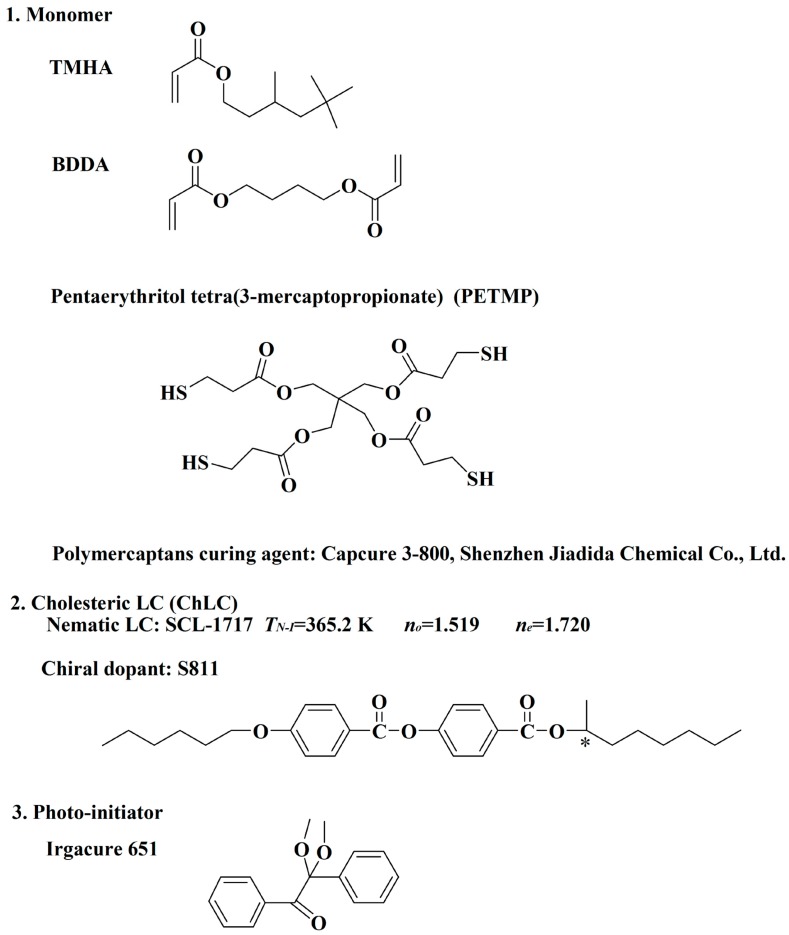
The chemical structures of the materials used. **1** is the monomer, **2** is the cholesteric LC, **3** is the photo-initiator.

Polymer-dispersed liquid crystal (PDLC) films were obtained by the PIPS process. The chemical structures and some physical properties of the materials are shown in Figure 1. In this study, the weight ratio between the acrylate monomer (TMHA) and diacrylate crosslinker monomer (BDDA) were fixed, but the concentration of chiral dopant S811 and feed ratio of the thiol crosslinking agents was changed (as shown in Table 1).

**Figure 2:**
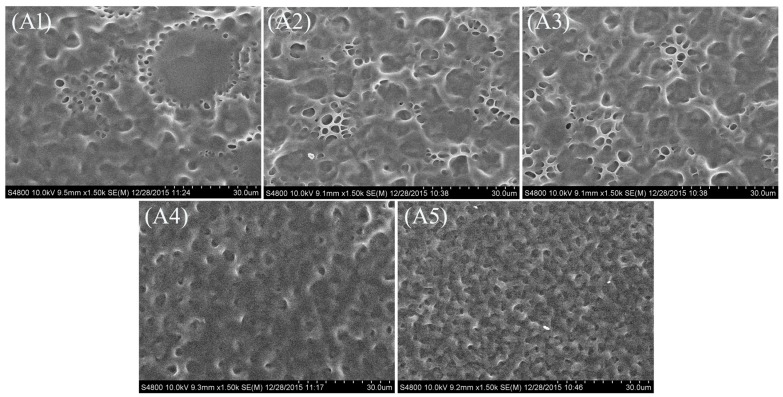
SEM micrographs of the polymer networks of the samples **A1**–**A5.**

Figure 2 shows the polymer networks of samples **A1**–**A5** with different amounts of S811. For samples **A1**–**A5**, it can be clearly seen that the LC domain size decreased with increasing the chiral dopant S811 content. It is well known the droplet size is controlled by the relative content of the photopolymerizable monomers and LC, the rate of polymerization, and some physical parameters such as the viscosity, rate of diffusion, and solubility of the LC in the polymer [29]. For a definite system, the relative content between photopolymerizable monomers and LC compound was fixed, but the concentration of chiral dopant was increased from sample **A1** to **A5**. From sample **A1** to sample **A5**, the content of S811 was 0, 1.0 wt %, 3.0 wt %, 5.0 wt %, 7.0 wt %, respectively. While increasing the S811 content caused the pitch to reduce, so the anchoring force from the polymer network on liquid crystals increases, and this resulted in the decreased LC droplet size.

**Figure 3:**
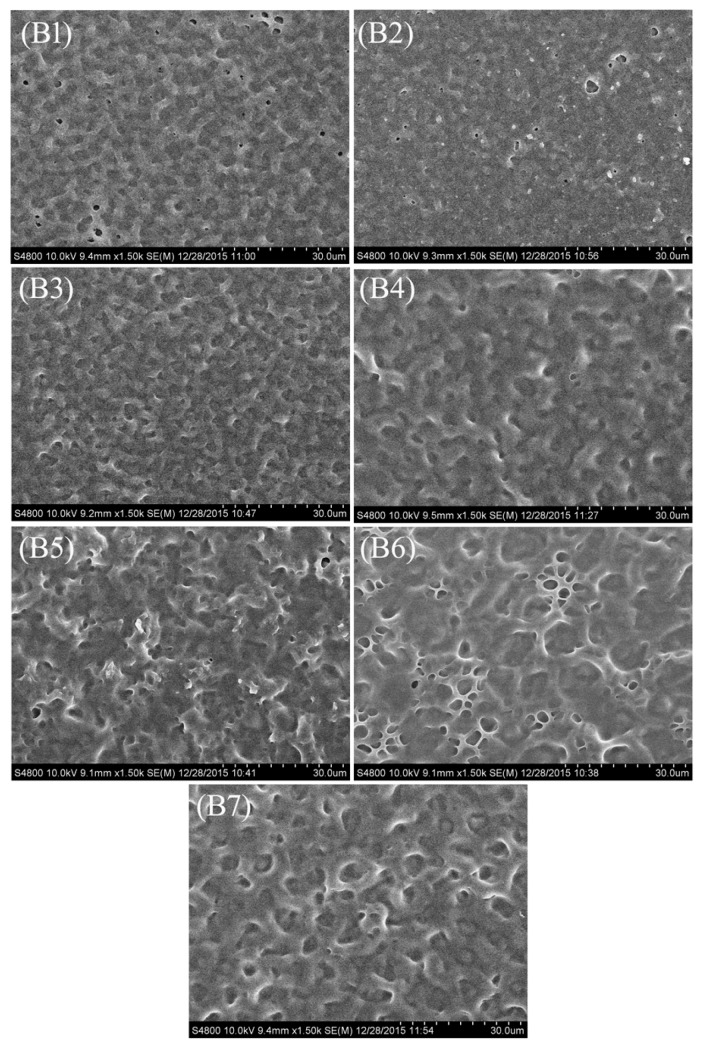
SEM micrographs of the polymer networks of the samples **B1**–**B7**.

Figure 3 shows the polymer networks of samples **B1**–**B7**. It can be seen that the mesh size (the LC droplets size) of the polymer network initially shows a slight decrease as the ratio of Capcure 3-800 increased from 0 wt % to 3.0 wt %, followed by a obviously increasing tendency as the concentration of Capcure 3-800 increased from 5.0 wt % to 9.0 wt %, and finally the LC droplets size showed a slight decreasing trend. This phenomenon may be due to the polymerization mechanism of thiol monomer. During the free radical polymerization process, the thiol radical does not polymerize with other thiol monomers. When the initiating thiol radicals are formed, the only means for the radicals to propagate is through addition to monomers with carbon-carbon double bonds [21,22]. In this study, we used the thiol monomer Capcure 3-800 and acrylate monomers. There was thus a competition between the thiol-acrylate and acrylate monomer polymerization reactions. The latter include homopolymerization and copolymerization reactions. In this situation, the crosslinking density increased and the LC domain sizes decreased with added Capcure 3-800 content into PDLC films. Then, due to the competition between different monomers, the crosslinking density changed dramatically. When the content of Capcure 3-800 was increased, accompanying with the decreased concentration of acrylate monomers, the carbon-carbon double bond was not sufficient enough for polymerization, so the LC droplet size increased with the increasing ratio of Capcure 3-800. The results indicate that the concentration of the polymercaptans curing agent showed a great influence on the mesh size of the polymer network.

**Figure 4:**
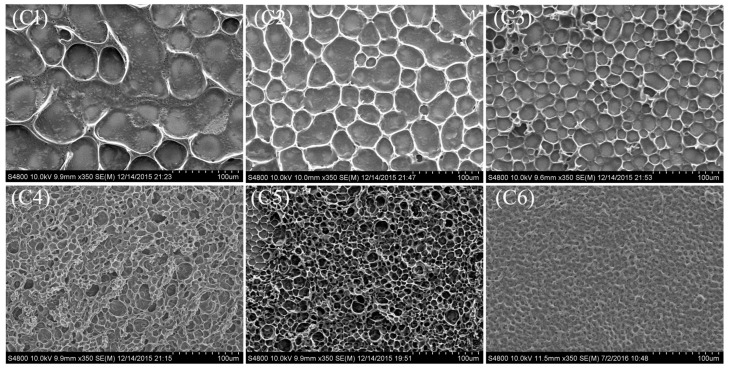
SEM micrographs of the polymer networks of the samples **C1**–**C6**.

Figure 4 shows the SEM photos of the morphologies of the PDLC films with different ratios of Capcure 3-800/PETMP. In the group C samples, acrylate monomers contained monofunctional acrylate monomer (TMHA) and bifunctional acrylate crosslinking monomer (BDDA). The weight ratio between TMHA and BDDA were fixed, and the relative weight ratio of thiol monomers Capcure 3-800 and pentaerythritol tetra (3-mercaptopropionate) (PETMP) were changed. From sample **C1** to sample **C6**, the LC domain size obviously decreases as the relative weight ratio of Capcure 3-800 increased from 0 wt % to 10.0 wt %. This phenomenon can be explained by the fact that the crosslinking density of PETMP is lower than that of Capcure 3-800, so the LC domain size would decrease as the Capcure 3-800 content increased. It is also shown that the polymer network of the PDLC film was significantly influenced by the relative ratios of the crosslinking agents.

### 2.2. Electro-Optical Properties of PDLC Films

**Figure 5:**
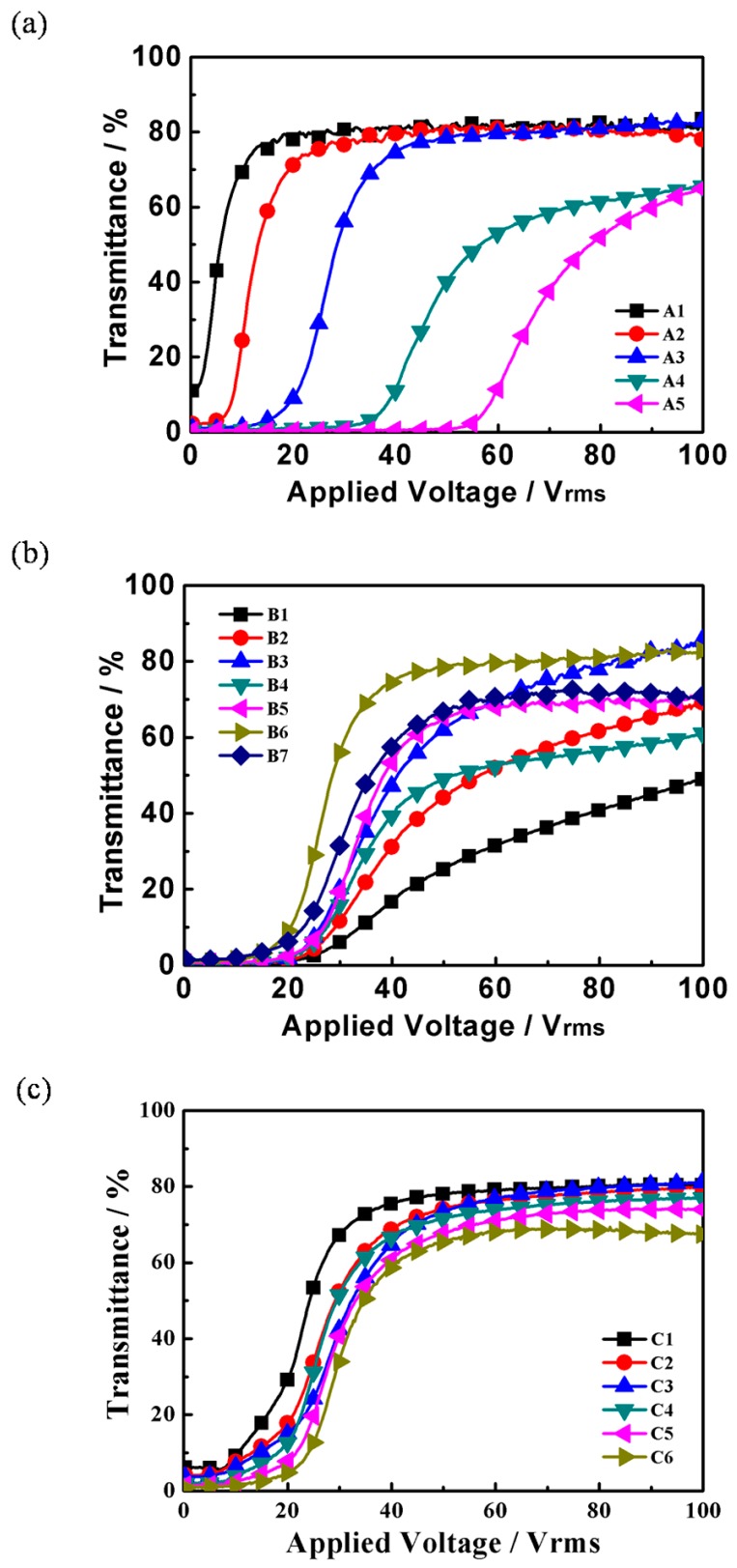
(**a**) The transmittance-applied voltage curves of group **A**; (**b**) the transmittance-applied voltage curves of group **B**; (**c**) the transmittance-applied voltage curves of group **C**.

Electro-optical properties are very important in the evaluation of PDLC films. As mentioned above, the content of thiol monomer and chiral dopant played important roles in the fabrication of the polymer network. Figure 5 show the transmittance-applied voltage curves of samples in groups **A**, **B** and **C**. With the applied voltage increased, the transmittance of the samples encompasses the initial off-state transmittance (*T_o_*) and the saturation transmittance (*T_s_*). As shown in Figure 5a,b, the sample **B6** which prepared with 9.0 wt % of Capcure 3-800 and 3.0 wt % S811 presented better properties than the others, the electro-optical properties is also significantly influenced by the ratio of Capcure 3-800/PETMP in Figure 5c.

The driving voltage, which includes the threshold voltage (*V_th_*) and the saturation voltage (*V_sat_*). is the major performance parameter of the PDLC films. The threshold voltage is defined as the electric field required for the transmittance to reach 10% of *T_s_*, while *V_sat_* is the transmittance required to reach 90% of *T_s_*. Generally, the threshold voltage *V_th_* is a linear function of the reciprocal of the size of the droplets *R* according to:

(1) Vth=dR[k(ω2−1)εo×Δε]12

**Figure 6:**
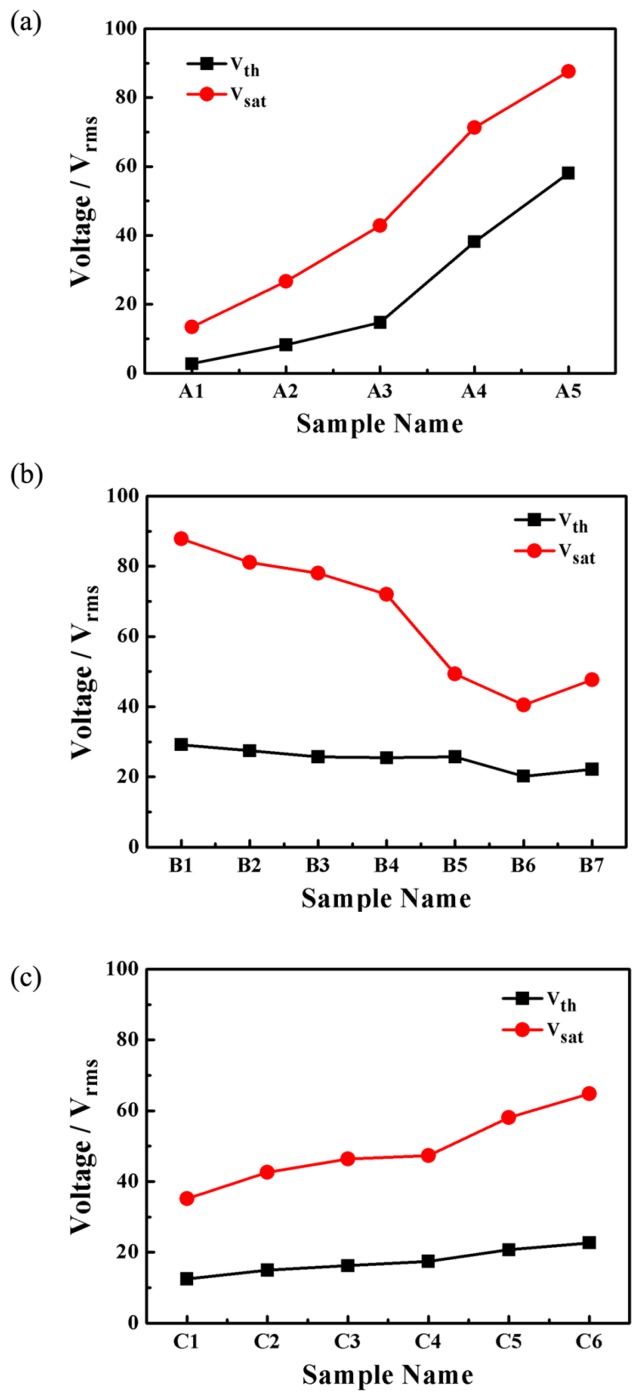
(**a**) Threshold voltage (*V_th_*) and saturation voltage (*V_sat_*) of samples **A1**–**A5**; (**b**) threshold voltage (*V_th_*) and saturation voltage (*V_sat_*) of samples **B1**–**B7**; (**c**) threshold voltage (*V_th_*) and saturation voltage (*V_sat_*) of samples **C1**–**C6**.

The equation holds for droplets with tangential alignment of the director, where *d*, *R*, *k*, *ω, ε_o_* and *Δε* represent film thickness, domain radius, the effective elastic constant, aspect ratio, vacuum permittivity and dielectric anisotropy of the LC, respectively [30,31]. *V_th_* is inversely proportional to the LC domain size. The *V_th_* and *V_sat_* values of the samples are summarized in Figure 6a where with the increase of contents of S811, *V_th_* gradually increased from 2.7 V to 58.1 V, and *V_sat_* increased from 13.4 V to 58.6 V. This was in good agreement with the morphologies of the PDLC films shown in Figure 2. Driving voltage is inversely proportional to the radius of LC droplets. Smaller LC droplet size leads to higher *V_th_* and *V_sat_*. For a definite system, the helical pitch (*P*) is defined as:

(2) $$P=\frac{1}{(HTP)\cdot x}$$

where *(HTP)* is the helical twisting power of the chiral dopant, *x* is the chiral dopant concentration. Increasing chiral dopant S811 content caused the helical pitch to decrease, so the twist elastic energy in the free energy of bipolar droplets was increased, and it needed a larger electric field to reorient the droplets, therefore, *V_th_* and *V_sat_* of the PDLC films increased with the increasing content of S811.

Figure 6b shows threshold voltage (*V_th_*) and saturation voltage (*V_sat_*) of samples **B1**–**B7**. With increasing the Capcure 3-800 content, *V_sat_* of samples **B1**–**B6** decreased dramatically from 87.8 V to 47.6 V and *V_th_* decreased from 29.2 V to 22.2 V. The *V_th_* and *V_sat_* of sample **B7** are higher than that of sample **B6** resulting from the decreasing size of the LC droplets in sample **B7**. This was in good agreement with the morphologies of the PDLC films in Figure 3. From Figure 3 it can be seen that with increasing the thiol monomer content, the mesh sizes presented a trend of initially increasing and decreasing in sequence, and finally they had a slight decreasing trend. Therefore, the size and shape of the LC droplets synergistically affect the electro-optical performance of the film.

Figure 6c shows threshold voltage (*V_th_*) and saturation voltage (*V_sat_*) of samples **C1**–**C6**, where it can be see that the driving voltage of the PDLC were strongly influenced by the relative weight ratio of crosslinking agents. With increasing concentration of Capcure 3-800, the *V_th_* and the *V_sat_* gradually increased from 12.4 V to 22.7 V and from 35.1 V to 64.8 V, respectively. This was in good agreement with the morphologies of the corresponding PDLC films (see Figure 4). *V_th_* and *V_sat_* of the PDLC films increased with the decreasing LC domain size.

The contrast ratio (*CR*) is an important measure of the electro-optical performance in a PDLC system. The contrast ratio (*CR*) is commonly known as the switching contrast ratio. It characterizes the difference between the transparent state and the opaque state, which is defined as:

(3) $$CR=T_{s}/T_{o}$$

**Figure 7:**
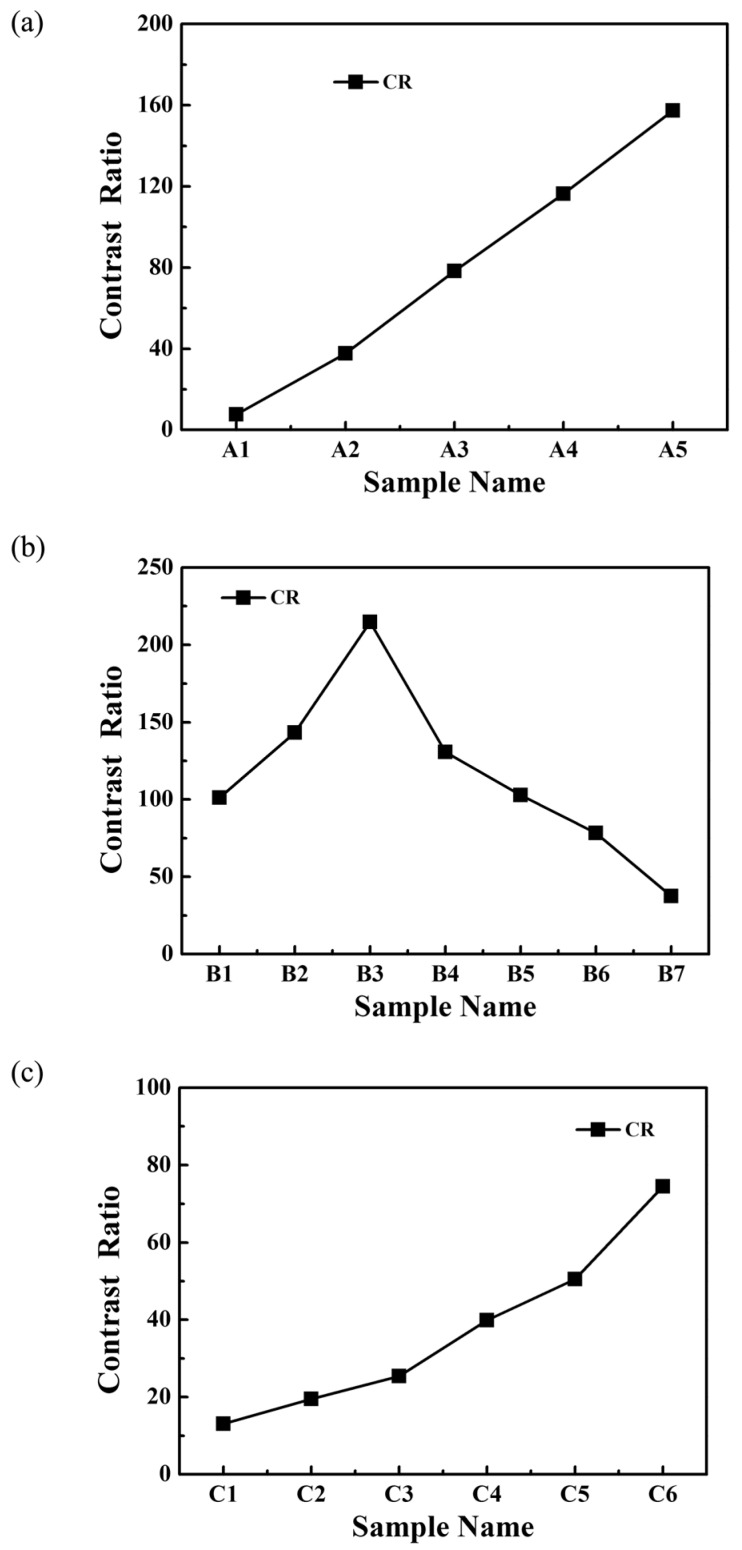
(**a**) The contrast ratio (CR) of samples **A1**–**A5**; (**b**) the contrast ratio (CR) of samples **B1**–**B7**; (**c**) the contrast ratio (CR) of samples **C1**–**C6**.

Figure 7 shows the contrast ratio of all samples. From Figure 7a, it can be seen that as the content of S811 increased, the *CR* increased because the off-state transmittance (*T_o_*) of the samples **A1**–**A5** decreased (Figure 5). Sample **A1** without S811 had the highest off-state transmittance (*T_o_*) value, but the low CR limits its practical application. Compared with the **A1**, the samples **A2**–**A5** of PDLC films have low *T_o_* (Figure 5a). With the increase of the S811 content, the off-state transmittance (*T_o_*) of the samples **A2**–**A5** decreased dramatically from 10.8% to 0.8%, while the saturation transmittance (*T_s_*) initially remained the same, followed by a slight decrease. The results show that the samples which were prepared with a content of chiral dopant S811 of no more than 3.0 wt % had a slightly lower contrast ratio, but an appropriate polymer network leading to a high on-state transmittance and low threshold voltage (*V_th_*), which are of great importance to the performance of the films. Hence, the optimum electro-optical properties of the PDLC films were obtained when the content of the chiral dopant was 3.0 wt %.

Figure 7b shows the *CR* of samples **B1**–**B7**. It is well-known that off-state transmittance (*T_o_*) of PDLC films depends greatly on LC domain size, with a not appropriate LC domain size results in insufficient scattering centers. As shown in Figure 7b, sample **B3** with 3.0 wt % Capcure 3-800 had the highest CR value due to the strong light scattering from uniform droplets. However, the low saturation transmittance limits its practical application. As shown in Figure 5b with increasing Capcure 3-800 content, the off-state transmittance (*T_o_*) decreased initially from samples **B1** to **B3**, and then increased from samples **B4** to **B7**, while the saturation transmittance (*T_s_*) of the samples increased gradually from 51.2% to 82.7% (**B1**–**B3**), then gradually increased from about 63.7% to 80.5% (**B4**–**B6**), finally followed by a slight decrease in sample **B7**. Compared to other samples, sample **B6** with 9.0 wt % Capcure 3-800 had a slightly lower contrast ratio, but it had appropriate polymer network leading to a higher on-state transmittance and lower threshold voltage (*V_th_*), which are of great importance to the performance of the films. From Figure 7c, it can be seen that as the relative weight ratio of Capcure 3-800 increases, the *CR* of the samples **C1**–**C6** increased from 13.1 to 74.4. The sample C6 with 9.0 wt % Capcure 3-800 had the highest *CR* value due to the strong light scattering from unifrom and smaller LC droplets (as shown in Figure 4). The results show that the properties of PDLC films based on thiol/acrylate system could be optimized by tuning the concentration of the chiral dopant and thiol monomers.

## 3. Materials and Methods

### 3.1. Materials

The nematic liquid crystal used in this study was SLC-1717 (Shijiazhuang Chengzhi Yonghua Display Material Co., Ltd. Shijiazhuang, China). The acrylate monomers is a mixture of 3,5,5-trimethelhexyl acrylate (TMHA, Sigma-Aldrich, 98%, Shanghai, China) and 1,4-butanedioldiacrylate (BDDA, Tokyo Chemical Industry Co., Ltd., 98%, Tokyo, Japan). The chiral dopant was S811 (Merck Co., Ltd., Shanghai, China). The pentaerythritol tetra-(3-mercaptopropionate) (PETMP, J&K Scientific Ltd., 95%, Beijing China) and polymercaptan curing agent (Capcure 3-800, Shenzhen Jiadida Chemical Co., Ltd., Shenzhen, China) were used as the co-polymerized monomers. The photoinitiator was Irgacure 651 (Ciba Geigy, Jingjiang Hongtai Chem. Co., Ltd., Jingjiang, China). The chemical structures and some physical properties of the materials are shown in Figure 1.

### 3.2. Sample Preparation

PDLC films were obtained by the PIPS process. At first, ultraviolet-curable monomer/crosslinking agent/chiral dopant/liquid crystal (LC) composite was prepared. Then, the composite was sandwiched between two pieces of indium tin oxide (ITO), conductive plastic films. The distance between the slides was controlled by a polyethylene terephtalate spacer of 20.0 ± 1.0 μm thickness. After the composite was irradiated by UV light (365 nm 35 W Hg lamp, PS135, UV Flood, Stockholm, Sweden) for 5 min at 25 °C, the UV intensity at the cell surface was about 13.5 mW/cm^2^ at 365.0 nm, a polymer network (polymer matrix) was formed in the composite from the crosslinking between the molecules of the photopolymerizable monomers. Thus, PDLC films were prepared. The compositions of the samples are listed in Table 1.

### 3.3. Morphological Analysis

The morphology of the samples was observed by scanning electron microscopy (SEM, S-4800, Hitachi, Tokyo, Japan). The films were first separated and dipped into hexane for 9 days at room temperature to extract the LC molecules, followed by drying for 10 h under vacuum. After the samples were sputtered with gold, the microstructure of the polymer network was observed under SEM.

### 3.4. Electro-Optical Measurements

The electro-optical property was measured using the LC device parameters tester (LCT-5016C, Changchun Liancheng Instrument Co., Ltd., Changchun, China). A halogen laser beam (λ = 560 nm) was used as the incident light source. The transmittance of the PDLC films was recorded by a photodiode. The response of the photodiode was monitored by a digital storage oscilloscope. An electric field square wave (100 Hz) was applied, and the distance between the PDLC film and photodiode was 300 mm. The collection angle of the transmitted intensity was about ±1° so that the forward scattering was mainly detected. The transmittance of air was normalized as 100%.

## 4. Conclusions

In summary, PDLC films based on thiol/acrylate monomers were prepared and characterized. The effects of the chiral dopant and crosslinking agents on the electro-optical properties of the PDLC films have been studied. The morphology of the PDLC films was strongly influenced by the chiral dopant S811 and polymercaptan curing agent Capcure 3-800. It was found that by adding appropriate concentrations of thiol monomer and chiral dopant into the PDLC system, lower driven voltages and higher contrast ratios were achieved. Compared with the PDLC films, S811 dopant resulted in a decrease in the off-state transmittance and an increase in light scattering. Due to the existence of competition between the thiol-acrylate and acrylate monomer polymerization reactions, the weight ratio among acrylate monomers, thiol monomer PETMP and the polymercaptan Capcure 3-800 showed great influence on the properties of the as-fabricated PDLC films. The results show that it is possible to tune the LC domain size and optimize the electro-optical properties of PDLC films by adjusting the chiral dopant and the weight ratio of thiol/acrylate.

## Figures and Tables

**Table 1:** The compositions of samples.

Sample	Monomers (20.0 wt %)		LC (80.0 wt %)
	Composition of Monomers		
Group **A**	TMHA + BDDAa/Capcure 3-800		SLC1717/S811
**A1**	11.0/9.0		80.0/-
**A2**	11.0/9.0		79.0/1.0
**A3**	11.0/9.0		77.0/3.0
**A4**	11.0/9.0		75.0/5.0
**A5**	11.0/9.0		73.0/7.0
Group **B**	TMHA + BDDA ^a^/Capcure 3-800		SLC1717/S811
**B1**	20.0/-		77.0/3.0
**B2**	19.0/1.0		77.0/3.0
**B3**	17.0/3.0		77.0/3.0
**B4**	15.0/5.0		77.0/3.0
**B5**	13.0/7.0		77.0/3.0
**B6**	11.0/9.0		77.0/3.0
**B7**	9.0/11.0		77.0/3.0
Group **C**	TMHA + BDDA ^a^	Capcure 3-800/PETMP	SLC1717/S811
**C1**	10.0	-/10.0	77.0/3.0
**C2**	10.0	2.0/8.0	77.0/3.0
**C3**	10.0	4.0/6.0	77.0/3.0
**C4**	10.0	6.0/4.0	77.0/3.0
**C5**	10.0	8.0/2.0	77.0/3.0
**C6**	10.0	10.0/-	77.0/3.0

^a^ monomer mixture (TMHA + BDDA): TMHA/BDDA = 4/1 (by wt %).
